# Developing appropriate environmental enrichment strategies for broiler breeders

**DOI:** 10.1038/s41598-025-89691-w

**Published:** 2025-03-21

**Authors:** Mary Baxter, Niamh E. O’Connell

**Affiliations:** Institute for Global Food Security, Queens University Belfast, 19 Chlorine Gardens, Belfast, BT9 5DL Northern Ireland

**Keywords:** Agricultural genetics, Animal behaviour

## Abstract

There is a recognised need for environmental enrichment strategies to be developed for broiler parent stock. We evaluated the use and tested preferences for three forms of enrichment in commercial broiler breeder housing: (1) perches (platform perches, round metal perches, round rubber-coated metal perches), (2) pecking enrichments (silver reflective gazing balls, multi-coloured reflective gazing balls, non-reflective gazing balls), and (3) dust baths (oat hulls, sawdust, 50:50 mix). Observations from video footage were conducted during early, peak and late lay of a 44 week production phase on one farm. There was a clear preference for platform perches over both round perches, and for rubber-coated perches over metal perches until late lay. Perching was highest at night but reduced over the cycle. We found a drop off in perching once 23 cm of linear space per bird had been reached, suggesting this represented comfortable maximum capacity. All pecking enrichments attracted interest, with no difference between the three types, although pecking rate reduced across the cycle. House litter was well maintained and dustbathing was widely observed throughout the house, making additional dustbathing areas largely superfluous on this farm. We suggest platform perches and suspended gazing balls to be suitable environmental enrichments for commercial breeder housing.

## Introduction

Increasing the complexity of poultry housing using environmental enrichment can have positive and sustained effects on welfare^1–3^. Recently, the resolve to improve the welfare of broiler chickens has intensified. Many European food companies have committed to only sourcing chicken from slower-growing breeds with access to enrichments, including a minimum 2 m of perch space and 2 pecking substrates per 1000 birds by 2026^4^. There has therefore been an increase in commercial interest in appropriate enrichment strategies, and a recognized need for this to be extended to the parent stock of broiler chickens: “broiler breeders”. As broiler breeders share the same genetic selection for rapid growth and high muscle mass as their offspring, up to 70% feed restriction is used in the EU to slow their development and reduce the obesity related health issues common in broiler chickens^5^. This allows them to reach sexual maturity and produce eggs, with a level of activity more closely resembling laying hens than broiler chickens. However, differing welfare concerns and housing systems mean it is likely they remain distinct from laying hens in their behavioural needs. Our understanding of modern broiler breeder preferences is limited. For example, a literature review on breeder enrichment found only four peer-reviewed papers published between 2000 and 2017, most of which focussed on the rearing period^6^. Therefore, a wider evidence base remains needed for broiler breeders to help inform future commercial housing standards.

Perches are a common environmental enrichment in poultry systems and satisfy an innate motivation for fowl to roost at night on the highest accessible surface^7,8^. There is an EU wide directive for all laying hens to have access to perches, with 15 cm per hen at a minimum height of 45 cm (Council Directive 1999/74/EC). The initial EU recommendation also covered broiler breeders^9^, however no uniform legislation is in place in or within member states. For example, Lower Saxony in Germany require at least half of broiler breeders in the house to have access to 20 cm perch length per bird, in addition to the raised nesting platform^5,10^. Switzerland is the only European country with country-wide legislation, requiring 14 cm of perch length per bird^11^, although there are various ways this is implemented^5^. Recently, the European Food Safety Authority (EFSA)^5^ made no recommendation for material, shape and diameter of perches for broiler breeders, citing a lack of research. It can be difficult to untangle the effects of height, material and shape in terms of bird preferences. Higher levels of perching in broiler breeders have been reported on wooden slats over wooden perches and mushroom-shaped (flat but rounded) plastic perches^12,13^, on steel feeder lines over lower plastic mushroom perches^14^, and on higher mushroom plastic perches over round steel, square steel and wooden perches^15^. More preference research is available for other poultry systems, with laying hens preferring high rounded perches that allow grasping over flat platform (grid) perches^8^ and broilers preferring platform perches that offer more stability^16,17^. Wider 45 mm perches also tend to illicit fewer balance adjustments in laying hens compared to thinner perches, which supports the widespread commercial use of 45 mm perches^18,19^. Although metal perches are common, EFSA^20^ ranked perches with rubber coating higher than plastic and metal, largely due to the expectation of rubber being soft, non-slippery and more thermally stable. Crucially, perches must also be designed with commercial installation in mind. Therefore, following discussion with farm and producer staff, we tested breeder preferences for three perch types that may satisfy roosting behaviour and could be practically installed in commercial housing: (1) 45 mm round metal bar perches, (2) 45 mm rubber-coated round metal bar perches, and (3) 16 cm wide platform perches made of plastic gridding. Both the 45 mm bars and plastic gridding were already in use in the breeder housing to hold feeder bulbs and for nesting platforms, respectively, making them simple to source and scalable. To avoid blocking nest access and provide birds with a satisfying height level, all perches were installed 53 cm above the nesting platform between wooden roof support posts, making them ~ 1 m above floor level.

Poultry have a sophisticated sense of vision and would naturally spend the majority of their time foraging and exploring their surroundings^21–23^. Commercial housing tends to be homogenous by nature and the introduction of visual or manipulable enrichments has the potential to reduce boredom and encourage birds to interact with their environment^24,25^. For broiler breeders specifically, installing enrichments on the floor level of the house may encourage a more even distribution of hens, which could reduce male-male competition and improve reproductive performance^26^. Poultry interest in various enrichments has been tested in laboratory and commercial settings, including moving light spots^27^, plastic chain^28^, toys^29^, string^25,30^, CDs^31^ and videos^32^. Chicks have shown an innate interest in bright, reflective surfaces, especially when they move^33–35^. Reflective enrichments may also combat habituation by offering a constantly novel and changing surface. Therefore, we chose to test 15 cm spherical “gazing balls”, which could be suspended from the house ceiling at breeder head height, as visually stimulating pecking enrichments. To explore whether a reflective or colourful surface made them more attractive and encouraged more sustained use, we compared three forms: (1) a silver reflective gazing ball, (2) a multi-coloured reflective gazing ball, and (3) a silver brushed steel gazing ball that was not reflective.

Lastly, we aimed to assess the value of introducing dust baths to commercial breeder housing. Dustbathing is a highly motivated behaviour in poultry and its deprivation is linked with frustration and reduced feather condition^36–38^. However, beyond considering dustbathing to be a behavioural need in poultry, little is known about how broiler breeders would make use of dust baths. To our knowledge, no studies have provided broiler breeders with dedicated dustbathing areas or tested their substrate preferences. In broiler housing, we found a preference for peat and oat hulls as dustbathing substrates over wood shavings, straw pellets and the house litter^39^. As oat hulls are a farming by-product and likely to be more suitable for industry compared to other friable materials, including peat and sand (Moy Park Ltd, personal communication), we proposed oat hulls as a suitable material for commercial housing. However, oat hulls are also a source of edible fibre. Due to the feed restriction that broiler breeders experience, it is possible that oat hulls would be used as a food source rather than dustbathing substrate. Therefore, to evaluate the use of dust baths in commercial housing and to determine whether oat hulls would primarily elicit feeding behaviour compared to a similar but non-nutritive material, we assessed the use of temporary dust baths containing: (1) oat hulls, (2) sawdust, which was similar in consistency and colour to oat hulls but non-nutritive, and (3) a 50:50 mix.

The purpose of this study was to develop effective strategies for introducing environmental complexity to commercial broiler breeder housing during the production phase. We aimed to investigate breeder preference for different forms of perch, pecking enrichment and dust bath that would be pragmatic for industry and provide breeders with additional opportunities for natural roosting, exploratory and dustbathing behaviours. We also monitored breeder health, welfare and productivity parameters to ensure that enrichments did not have a negative impact.

## Materials and methods

All methods described in this paper were approved by the Queen’s University Belfast Faculty of Medicine, Health and Life Sciences Research Ethics Committee (reference number MHLS 22_52). All methods were performed in accordance with relevant guidelines and regulations, and are reported according to the ARRIVE guidelines.

### Animals and housing

This study was conducted between March and December 2022 on a commercial broiler breeder farm in Northern Ireland associated with Moy Park Ltd. Informed consent was provided by the farmer. At 19 weeks of age, a total of 7950 Ross 308 parent-stock broilers, “broiler breeders”, were placed in each of four identical houses, with approximately 91% females and 9% males. Broilers were expected to come into lay with 5% egg production in week 23, with peak egg production expected around week 29. Broiler breeders were cleared for slaughter at week 63.

**Fig. 1:**
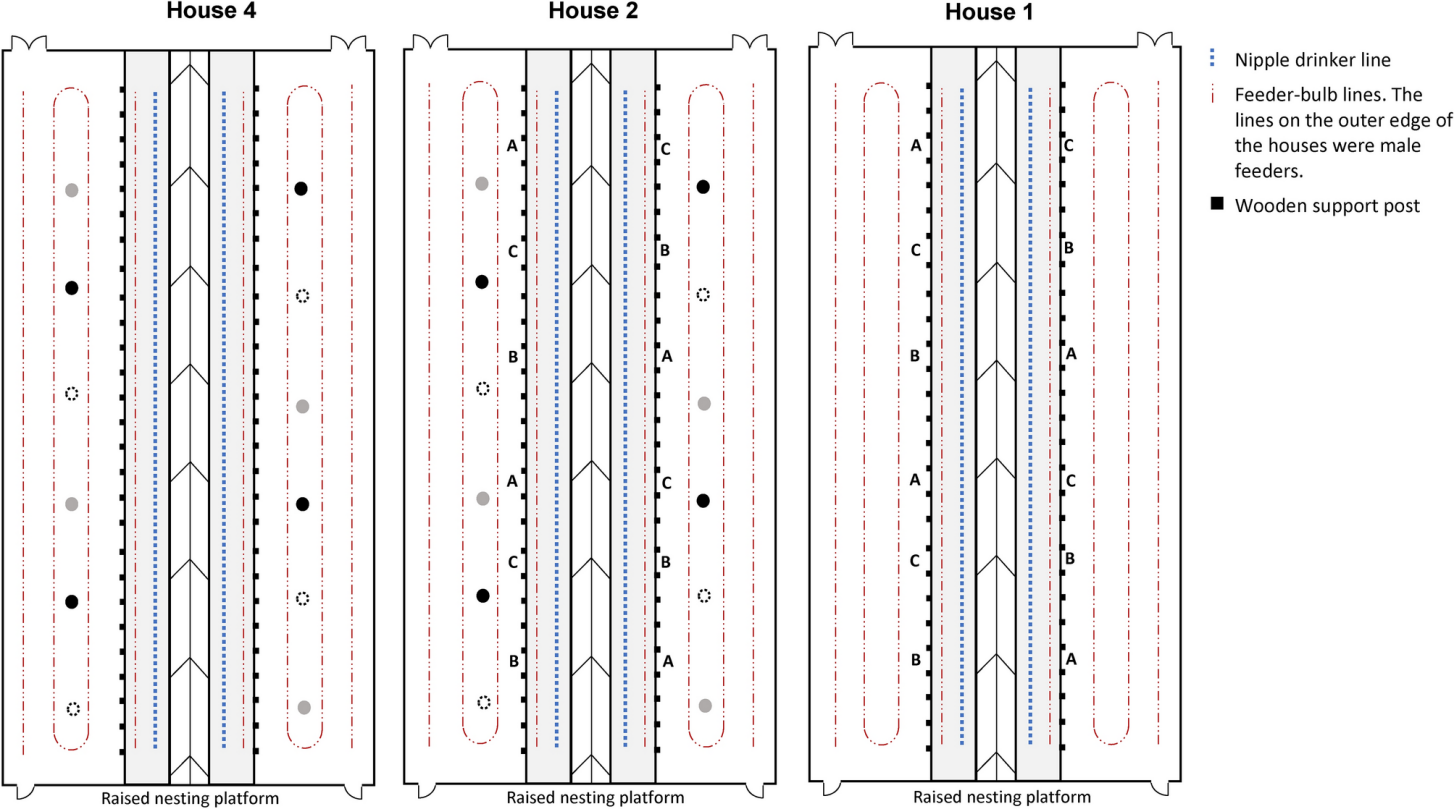
Schematic of three of the four houses involved in the study to demonstrate enrichment placement (not to scale). House 3 was used as an unenriched control and had the same layout as the remaining houses. House 1 contained perches only, either platform perches (**A**), metal bar perches (**B**) or rubber-coated bar perches (**C**). House 2 contained perches and suspended pecking objects, either reflective gazing balls (grey circles), non-reflective gazing balls (black circles) or multi-coloured gazing balls (white circles with dashed line). House 4 contained gazing balls only. Feeder lines were present on the floor and one on the raised nesting platform. One drinker line was present on the raised platform. Nest boxes ran along the central line of the house.

The four wood-framed houses (98 m × 14 m) followed the same floor plan with an open deep litter area and a raised central platform where the nest boxes were located (Fig. 1). The platform, made of plastic gridding with 2 × 4 cm holes, was raised 60 cm above ground level. Broilers were unable to access the area beneath the platform but could cross from one half of the house to the other via a 2 m opening at one end of the house or by flying across, although a gable roof and anti-perching lines on top of the nesting areas largely prevented this. Wooden support pillars ran floor to ceiling along the edge of the platform area, 3 m apart.

The floor area was bedded with 10 cm deep woodshavings at the start of the production cycle, with additional woodshavings distributed to maintain litter at the farmer’s discretion. The farmer also provided a number of plastic wrapped woodshavings bales at intervals (approximately weekly) to be pecked open by the birds.

Feed was provided via bell feeders, with a higher feeder line for males on the two outer edges of the house, two female feeding lines on the floor and one on top of the platform area (Fig. 1). Birds were fed once per day, with feed always released half an hour after the lights came on in the morning. Ad libitum water was provided through one nipple drinker line on the platform area on each side of the house (Fig. 1).

There were no windows and artificial light was provided with LED lamps. From placement until point of lay (POL, ~ 22 weeks), 8 h of 20–30% light intensity was provided from 05 00 h until 13 00 h in houses 2 and 4, and from 05 30 h until 13 30 h in houses 1 and 3. The lighting times in H2/H4 and H1/H3 varied due to feeding mechanisms being limited to two houses at a time and lights coming on half an hour before feed was released. From POL until 25 weeks, 11 h of light was provided from 05 00/05 30 h until approximately 16 00/16 30 h, with light intensity gradually increasing to 100%. From 25 weeks, the light period was increased to its maximum of 13 h, from 05 00/05 30 until 18 00/18 30 h.

### Treatments

**Fig. 2:**
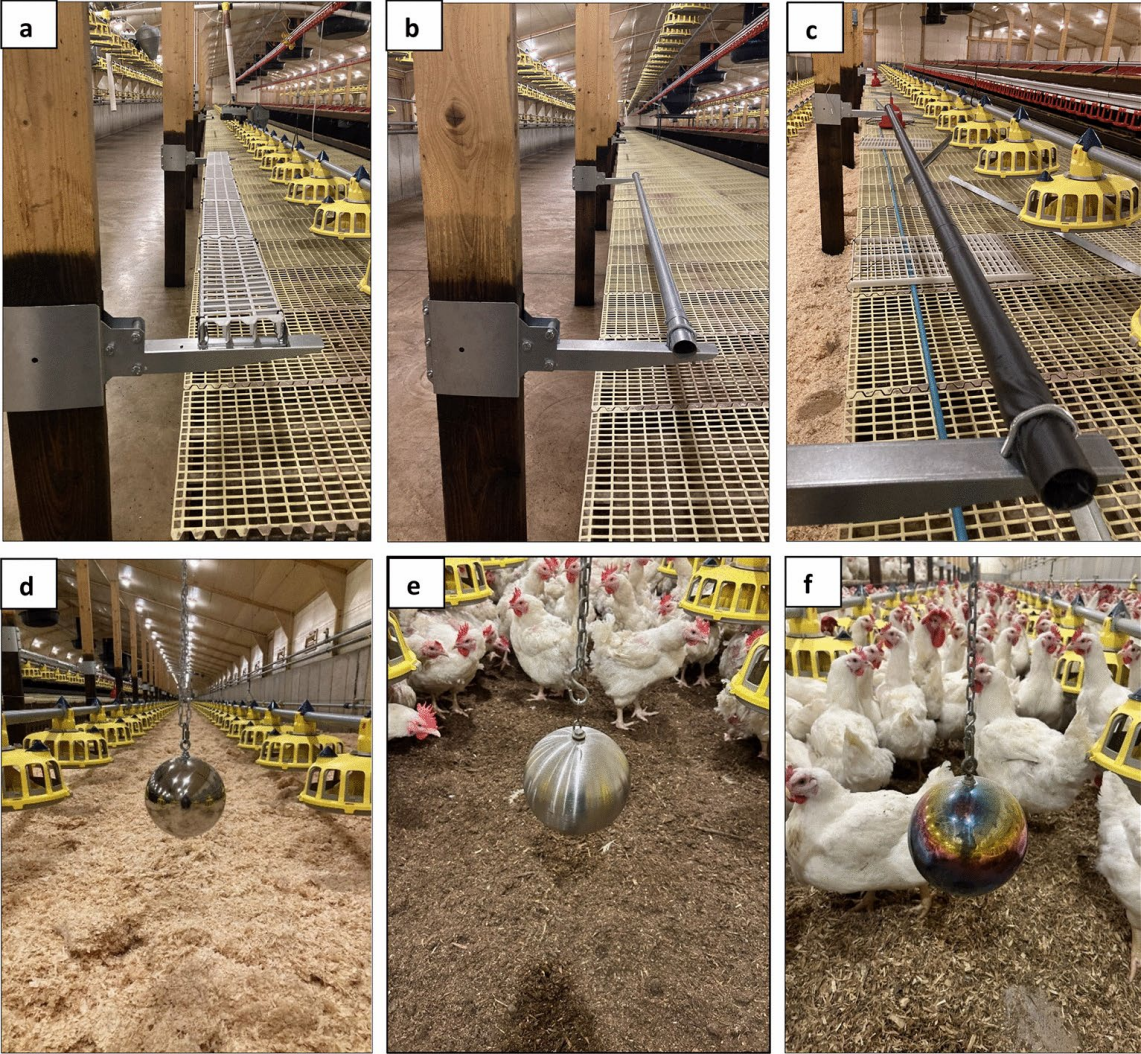
The different enrichments used in this study to identify which forms attract the most use from broiler breeders. Three perch types were installed: (**a**) platform perches, (**b**) metal bar perches and (**c**) rubber coated metal bars. Three forms of gazing ball as a pecking object were also installed: (**d**) reflective gazing ball, (**e**) non-reflective gazing ball and (**f**) multi-coloured reflective gazing ball.

Preferences for three forms of enrichment were tested during this study (Figs. 1, 2): (1) perches, (2) pecking enrichments, and (3) dust baths. To also monitor the effect of increasing environmental complexity on productivity, they were separated into three treatment houses with one additional house serving as a control (Fig. 1). House 1 contained only the three forms of perch, House 2 contained mixed enrichment with all forms of both perches and pecking objects, House 3 contained no enrichment and was used as an unenriched control house, and House 4 contained only the three forms of pecking object. All perches and gazing balls were installed before the birds arrived. At the farmers request, pecking objects were lowered to head height 3 days after the last house was stocked with birds to prevent disturbing their initial settling period. Dustbathing material was only presented on observation days, to limit maintenance for the farm staff.

The perches installed in House 1 and House 2 were three metres long and were fitted between the wooden support posts that ran along the edge of the raised nesting platform. At the farmer’s request, they were attached to the pillars with a steel arm that hinged upwards, allowing them to be raised out of the way and pushed flat against the pillars for cleaning (Fig. 2a–c). Perches were 53 cm above the platform and midway between the platform edge and the feeder line that ran along the nesting platform, 41 cm from the edge of the platform. Three forms of perches were installed, with six perches (two of each type) evenly spaced among the pillars on both sides of the house, giving a total of 12 perches per house. This provided 36 m of perch space per house, 4.5 m per 1000 birds. The three forms of perch were (1) platform perch made of plastic gridding, (2) round metal bar perch and (3) rubber-coated round metal bar perch. Platform perches were 16 cm wide and round perches had a 45 mm diameter. The rubber-coated bar perches consisted of the same 45 mm bars wrapped in butane rubber matting (Fig. 2c). The location of each perch type differed on each side of the house (Fig. 1).

House 2 and House 4 contained three forms of pecking enrichment (Fig. 2d–f): (1) a 15 cm diameter reflective stainless steel gazing ball (HomDSim, Amazon, London, UK), (2) a 15 cm diameter multi-coloured stainless steel gazing ball (GDGlobal, Amazon, London, UK), and (3) a 15 cm diameter non-reflective brushed stainless steel gazing ball (Köhko, Amazon, London, UK). Two of each form were suspended on each side of the house; a total of six per side and 12 gazing balls per house. The gazing balls were suspended from wooden ceiling joists at bird head height, approximately 40 cm from the litter, using metal chain. They were initially very light and so were filled with water before being hung to avoid excessive swinging.

Dustbathing material preference tests were also conducted in House 2 (the mixed enrichment house) once per week during peak and late lay (more details in Measurements). Plastic rectangle dustbathing areas were used to present each material and measured 1.2 m long, 60 cm wide and 15 cm deep. The rectangles had no base and were placed directly onto the litter with approximately 10 cm depth of substrate. The three dustbathing materials tested were (1) processed oat hulls, (2) sawdust, (3) oat hulls + sawdust. Sawdust and oat hulls had a similar fine consistency and light yellow colour. Oat hulls were considered to be a source of fibre and safe for broilers to eat, having been used before as a dustbathing substrate^40^ and food additive (e.g. ^41^). The oat hulls + sawdust mixture consisted of 50% of each material. Both oat hulls and sawdust underwent treatment with propionic acid during processing (mixed 1 part acid and 4 parts water) for biosecurity before they were brought into the house. Propionic acid typically has an odour, however this was not detectable to research staff during the study.

### Measurements

All data collection was performed by the same observer. Intra-rater reliability was not determined. The farm was visited twice per week for 4 weeks in three phases of production, early (weeks 24 to 27), peak (weeks 28 to 31) and late (weeks 43 to 46). Video footage of perches, pecking objects and unenriched areas was taken using infra-red enabled body cameras (Boblov, Shenzhen Lvyouyou Technology Co. Ltd, Shenzhen, CN) attached to external battery packs (Belkin, El Segundo, California) and mounted temporarily on the wall of the house. Footage of dustbathing areas was taken using Camileo X-Sports cameras (Toshiba, Surrey, UK) and GeeKam action cameras (Shenzhen Bodalong Technology Co., Ltd, Guangdong, CN) mounted on the house wall.

#### Perching behaviour

##### Perch occupancy

In both houses containing perches, one randomly selected perch of each type was selected on each side of the house. The cameras began filming before 08 30 h on Observation Day 1. The observer then left the house and returned to remove the cameras at 09 00 h the following day, Observation Day 2. This gave 24 h of footage of 12 perches per observation week; one of each form of perch (platform, round metal and round rubber-coated) on each of two sides of two houses. Perching behaviour was assessed hourly across the 24-h period. On the hour, from 10 00 h to 09 00 h, the number of broilers on each perch type was recorded (n = 24 observations per perch, per week; N = 3456).

##### Movement under perches

To determine whether the movement of breeders from the floor onto the nesting platform or from the nesting platform to the floor was affected by the presence of a perch at the edge of the nesting platform, we recorded the movement of broilers underneath perches and in the neighbouring empty section in treatment houses. During observation weeks chosen from early (weeks 24 and 25), peak (weeks 30 and 31) and late lay (weeks 44 and 46), one randomly selected perch of each type on the left and on the right of each house were used. For a 5 min focal period at 08 00 h, 12 00 h and 16 00 h, the number of broilers jumping onto and off the nesting platform area was recorded for the section with a perch and the neighbouring section without one (N = 216). The mean of the three focal periods were averaged to give one score per day (N = 72).

#### Pecking object preference

On the morning of Observation Day 2, cameras were transferred to record pecking enrichments in the two houses containing gazing balls for two hours from 10 00 h to 12 00 h. Three randomly selected gazing balls, one of each type, were recorded on each side of the two houses. The gazing ball chosen out of the two available was random but consistent between the two houses. This gave a total of 6 pecking objects recorded in each house each observation week. Due to missing footage for weeks 24, 26 and 43, the number of pecks directed at each recorded gazing ball were observed in weeks 25, 27- 31 and 44–46 over five-minute focal periods at 10 15 h and 11 15 h (N = 216). The number of pecks over the two focal periods was averaged for analysis to give one score per day (N = 108).

#### Dustbathing material preference

Due to concerns that dustbathing areas would risk increasing floor eggs during early lay, dustbathing tests were only performed during peak and late lay. To avoid additional work for farm staff, these dustbathing areas were also only filled by research staff and observed for ∼1 h to determine any initial differences in use. In weeks 28–31 and 43–46, six dustbathing rectangles were placed in House 2 (the house with mixed enrichments) on the morning of Observation Day 2, three on each side of the house. They were filled to a depth of approximately 10 cm with either oat hulls, sawdust or a 50:50 mix of oat hulls + sawdust. The placement of each substrate was rotated each week. Six cameras attached to the side of the house wall were set up to record all dustbathing areas simultaneously to the filming of pecking objects (starting at approximately 10 00 h) for a period of 1.5 h. After a 5 min settling period, dustbathing behaviour was assessed using scan sampling. Scan samples were performed at 5, 20, 35 and 50 min. For each scan, the number of broiler breeders inside the dustbathing areas were counted. They were recorded as male or female, and their behaviour was then classified over a 10 s period as foraging, dustbathing or other (e.g. eating, standing, sitting inactive). As in^40^, foraging was defined as “scratching and pecking at the substrate from a standing or walking position” and dustbathing was defined as “a bird was performing classic vertical wing shakes, and/or clearly covered in substrate and performing side-rubs or prone leg scratches”.

#### Overall flock measures

Each house contained one enrichment condition: perches only, perches and gazing balls, gazing balls only or unenriched control. As this study was primarily a preference test of different enrichment types (spread between different houses) and conducted over an extended period, it was not possible to perform multiple repetitions and therefore treatment effects are confounded with house effects for any measures taken from the whole house. However, it was important that increasing the complexity of the houses did not have a clear negative impact on broiler breeder health and productivity. Therefore, we took preliminary measures of feather score during early, peak and late lay in each house. Production records of mortality, egg production and % floor eggs were taken by farm staff. Production records were monitored as standard to ensure they stayed within standard commercial levels and descriptives are presented.

Feather condition was assessed on three occasions during the production cycle: (1) in week 25 as birds began lay, (2) in week 31 following peak lay, (3) in week 46 as lay dropped off. This was performed in a method adapted for on-farm assessment while avoiding penning birds. In 25 areas on the left of the house and 25 areas on the right, 2 birds per area were selected using random coordinates on a Perspex grid that was held up by the observer. The feather score of 100 females per house was recorded using a 6-point scoring system for each of five body areas: the head, neck, back, wing and tail. For each area, a score of 0–5 was given (score 0: no visible feather or skin damage; score 1: less than 10% feather damage or loss; score 2: between 10 and 50% feather damage or loss; score 3: more than 50% feather damage or loss; score 4: between 10 and 50% feather damage or loss with skin lesion[s]; score 5: more than 50% feather damage or loss with skin lesion[s]). Scores from the 5 areas were summed to result in a total feather coverage score from 0 to 25, with 25 being the worst feather coverage condition (bird completely denuded including tissue damage; based on^42,43^. A floor egg was classified as any egg laid on the litter of the house. Mortality included culls and those found dead.

## Statistical analysis

All data were analysed using SPSS v29. Estimated marginal means (EMM), standard error (SE), and standard deviation (±) are reported as appropriate. Perch occupancy data, collected hourly over 24-h periods across three time periods (early, peak and late lay; N = 3456), were averaged to provide the mean number of broilers perching daily on each perch (N = 144). Final N = 138 due to missing data in week 45 from equipment error. A linear mixed model with perch type (platform, round metal or round rubber-coated), time period and their interaction as fixed effects was used, with house as a random effect. Model residuals were normally distributed. Simple effects analysis was used to investigate significant interactions and pairwise post-hoc comparisons were performed with Bonferroni correction. Perch occupancy was also separated to day (04 00 h to 16 00 h) and nighttime (17 00 h to 03 00 h) perching for descriptive data. For movement under perches data (N = 72), a linear mixed model examined the impact of the presence or type of perch, time period, and their interaction on the average number of breeders jumping onto the nesting platform. House was included as a random effect. This approach was also used to assess the impact of the presence of a perch on breeders jumping down on to the floor, however a lack of convergence in the model meant that a fixed effects only model was used to evaluate the impact of perch type.

Data for weeks 24 and 26 were missing for pecking object data. Therefore, the number of pecks directed at each recorded gazing ball was observed in weeks 25 and 27 for early lay (N = 22), 28–31 for peak lay (N = 44) and 44–46 for late lay (N = 33). A linear mixed model robust to unequal sample sizes was used with type (reflective, multi-coloured or non-reflective), time period and their interaction as fixed effects and house as a random effect. Dustbathing scan samples were averaged per substrate video analysed (N = 44). Male and female behaviour data were transformed into percentages to give the proportion of birds performing dustbathing, foraging, or other activities. Kruskall-Wallis H tests with Bonferroni corrected pairwise comparisons were used for analysis.

Overall flock measures were taken to determine the effect of enriching the breeder’s environment. As these measures were only taken in four houses each containing one treatment, cautious interpretation of the results has been made and descriptive statistics are presented. Feather scores were recorded for 100 birds per treatment house in each of the three time periods (N = 1200). Production data including % floor eggs, egg production and mortality were taken by farm staff as standard.

## Results

### Perching behaviour

#### Perch occupancy

**Fig. 3:**
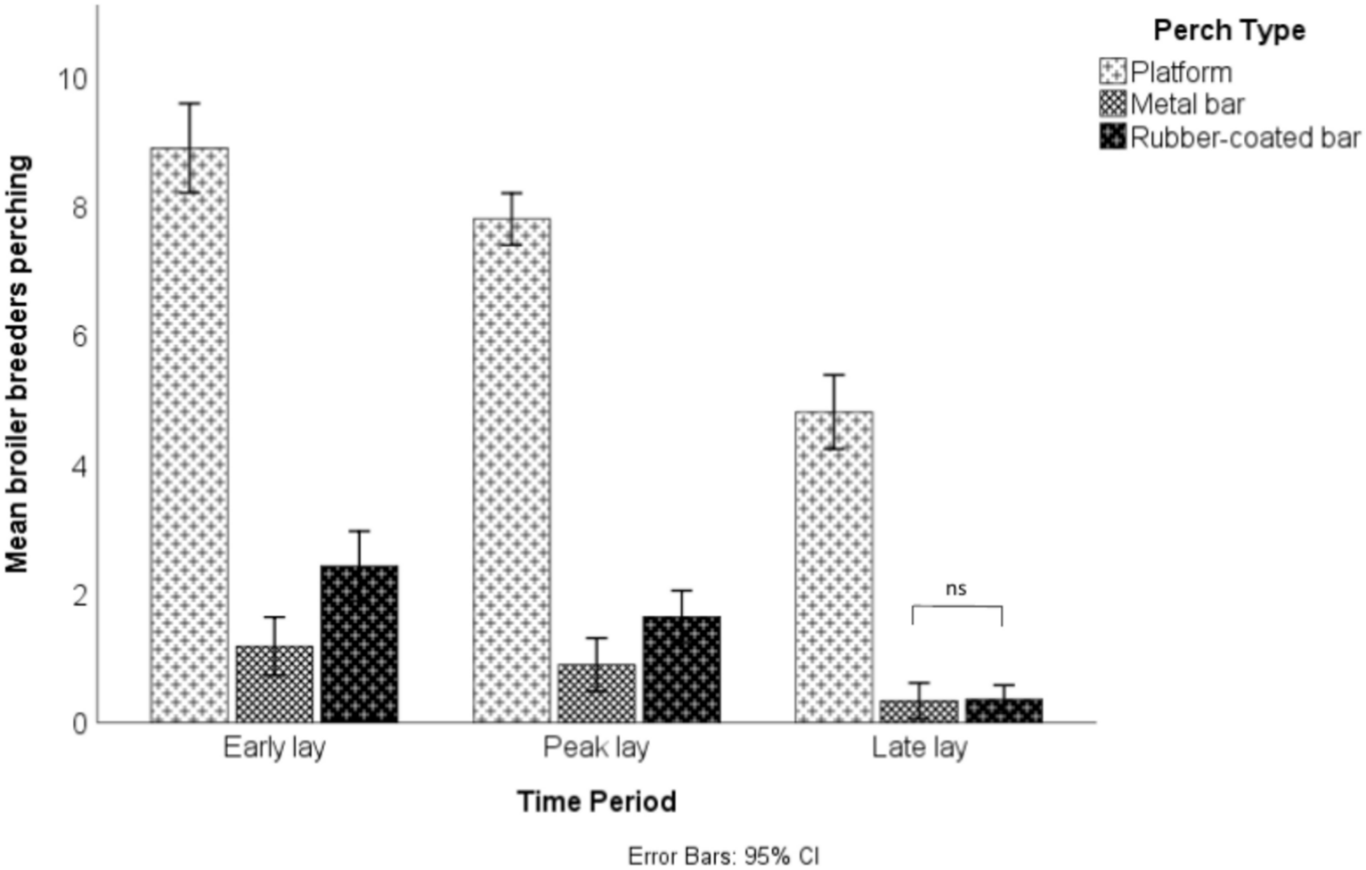
Perching levels in early (weeks 24–27), peak (weeks 28–31) and late lay (43–46) on platform perches, metal bar perches on rubber-coated bar perches in a commercial broiler breeder house. ns indicates a non-significant p > 0.05 result for simple effects analysis. All remaining simple effects comparisons between perch types, within time periods, were significant (p < 0.05).

There was a significant interaction between perch type (platform, round metal or round rubber-coated) and time period (early, peak or late lay) on the average number of broiler breeders perching across a 24-h period (F_4,128_ = 14.89, p < 0.001; Fig. 3). This indicated that the effect of perch type on occupancy was dependent on when the measures were taken across the cycle. Simple effects analysis revealed that there was a significant difference between all perch types in early and peak lay (p < 0.05), with the highest levels of perching seen on platform perches, followed by round rubber-coated perches and the least seen on round metal perches. However, in late lay the difference between the two least used perches was reduced, with no significant difference in perching levels between rubber-coated bars and metal bars (p > 0.05).

There was an overall mean (M) of 7.27 ± 1.99 breeders perching on the platform perches, 0.83 ± 0.79 on the metal bar perches and 1.53 ± 1.13 on the rubber-coated metal bars. A reduction in perching was seen across the production cycle, although there was a relatively wide spread of data in each time period due to the difference in perch occupancy between the perch types and day/night perching (early lay M = 4.17 ± 3.57, peak lay M = 3.45 ± 3.21, late lay M = 1.84 ± 2.23).

**Fig. 4:**
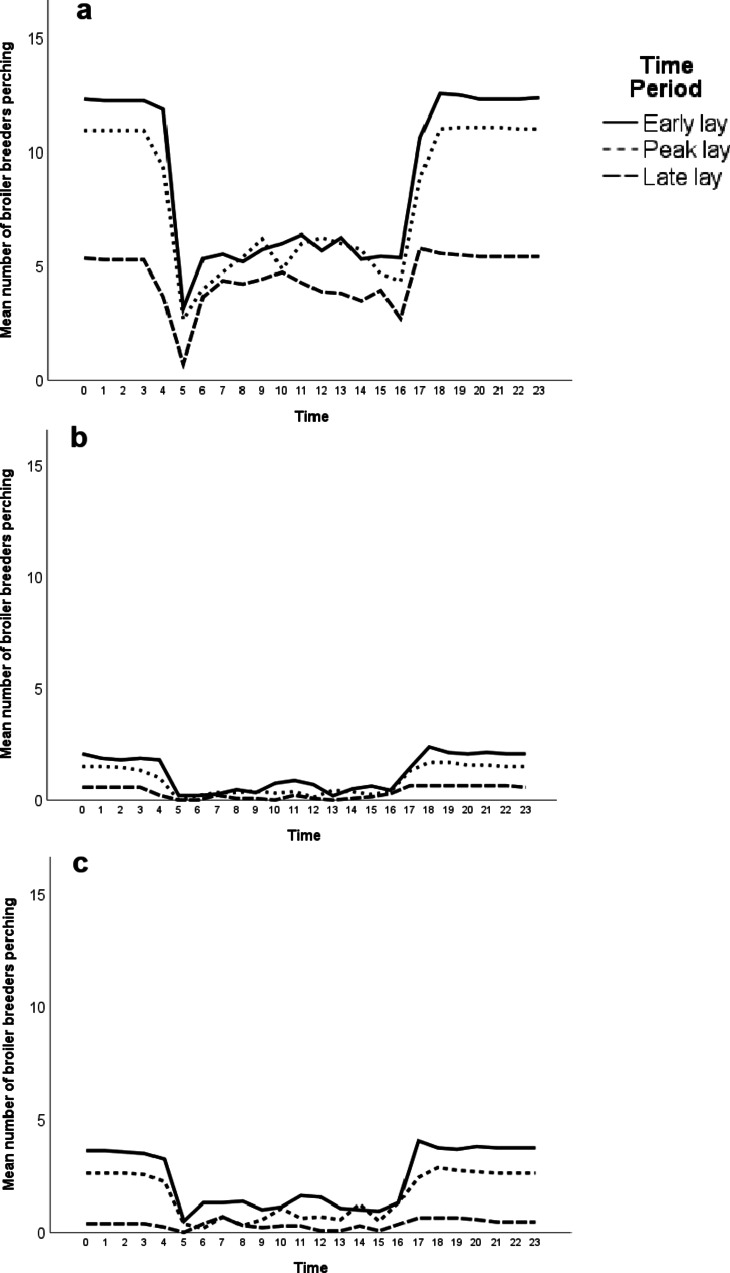
Average number of broiler breeders perching on three types of perch in a commercial house. Broilers were observed perching on platform perches (**a**), round metal perches (**b**) or rubber-coated perches (**c**) over 24 h periods in three time periods across the production cycle. The time period consisted of either early lay (weeks 24–27), peak lay (weeks 28–31) or late lay (weeks 43–46). Lights came on in the houses at around 04 00 h and turned off at around 17 00 h.

**Fig. 5:**
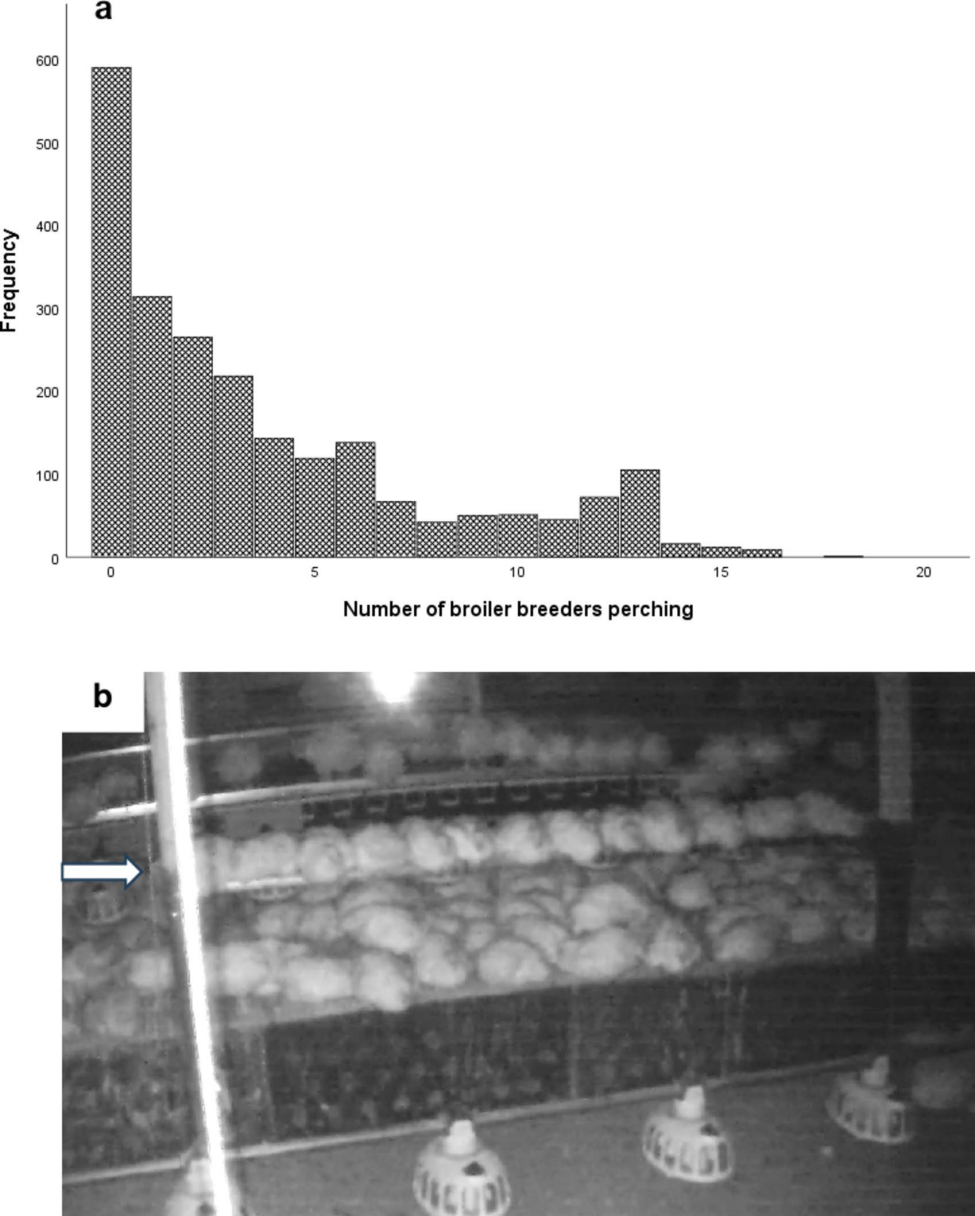
(**a**) Histogram of raw perch occupancy data for all perches. The maximum number of broilers recorded on a perch was 18 (on the platform perch). However, there were fewer incidences of more than 13 broilers perching at any one time, suggesting that the perches were becoming too full to easily accommodate more birds after this point. (**b**) An image of 13 broilers perching on a 3 m platform perch fitted between two wooden roof support posts. This occupancy of 13 birds allows 23 cm of linear space per bird. We considered this to be maximum comfortable occupancy. Broilers can also be seen resting on the raised nesting platform area and perching on the drinking line visible behind the platform perch.

As expected, for all time periods and perch types, the perches were used more at night than during the day, with this difference clearest in platform perches (Fig. 4a–c). Broilers were fed after lights on in the morning so all perches were typically vacated once the light levels began increasing. Perch occupancy typically increased and stabilised around one hour after lights off in the evening. The maximum number of broilers recorded on the round metal perches was 6 birds, on the round rubber-coated perches was 9 birds and on the platform perches was 18 birds. However, there was a drop off in the frequency of more than 13 broilers perching at any one time (Fig. 5a), suggesting that the perches had become too full to easily accommodate more broilers. At 13 broilers, the perch was visually full when all the birds were seated (Fig. 5b). This level of occupancy provided each roosting broiler with 23 cm of linear space. Using this level of maximum occupancy, nighttime perching on platform perches saw an average of 9.62 birds or 74% occupancy per perch, with maximum occupancy reached or exceeded in 27% of hourly nighttime observations. Daytime perching on platform perches saw an average of 5.04 birds or 39% occupancy, with maximum occupancy reached or exceeded in 2% of observations. On round metal perches, there were an average 0.35 birds perching (3% occupancy) during the day and 1.39 birds perching (11% occupancy) during the night. On round rubber-coated perches, there were an average of 0.82 birds (6% occupancy) during the day and 2.39 birds (18% occupancy) at night. Maximum occupancy was never reached on metal bars or rubber-coated bars during day or night perching.

#### Movement under perches

There was no significant difference in the number of broiler breeders jumping up onto the nesting platform when there was a perch present compared to no perch (N = 144; p > 0.05). An average of 10.49 ± 4.49 broiler breeders jumped onto the nesting platform during 5 min focal periods when there was a perch above them, and an average of 11.50 ± 4.66 when there was no perch. There was similarly no effect on the movement of breeders from the nesting platform onto the floor, with no significant difference in the number of broilers jumping down in the presence of a perch (M = 10.41 ± 4.47) or no perch (10.73 ± 6.16; p > 0.05). Time period did have a significant impact on the average number of birds moving up onto the nesting platform (F_2,137_ = 15.62, p < 0.001) and down onto the floor (F_2,137_ = 13.00, p < 0.001). There were significantly fewer broilers jumping up onto the nesting platform in late lay (EMM = 8.47, SE = 1.44) compared to early (EMM = 13.04, SE = 1.44) and peak lay (EMM = 11.46, SE = 1.44; p < 0.001). The same pattern was seen in those jumping down in early (EMM = 13.19, SE = 0.81), peak (EMM = 10.54, SE = 0.81) and late lay (EMM = 8.04, SE = 0.81), with post hoc comparisons significant for all pairs of time periods (early vs peak, early vs late, and peak vs late; p < 0.05). There was no interaction between the presence of a perch and time period (p > 0.05). We also explored whether the type of perch made a difference to the movement of birds underneath them. Perch type did not have an effect on the average number of breeders jumping up onto the nesting platform (platform M = 10.94 ± 5.20, round metal M = 9.63 ± 3.70, round rubber-coated M = 10.89 ± 4.51) or down from the nesting platform onto the floor (platform M = 11.22 ± 7.45, round metal M = 10.54 ± 4.44, round rubber-coated M = 9.49 ± 3.92; p > 0.05). There was also no interaction between perch type and time period (p > 0.05).

### Pecking object preference

**Fig. 6:**
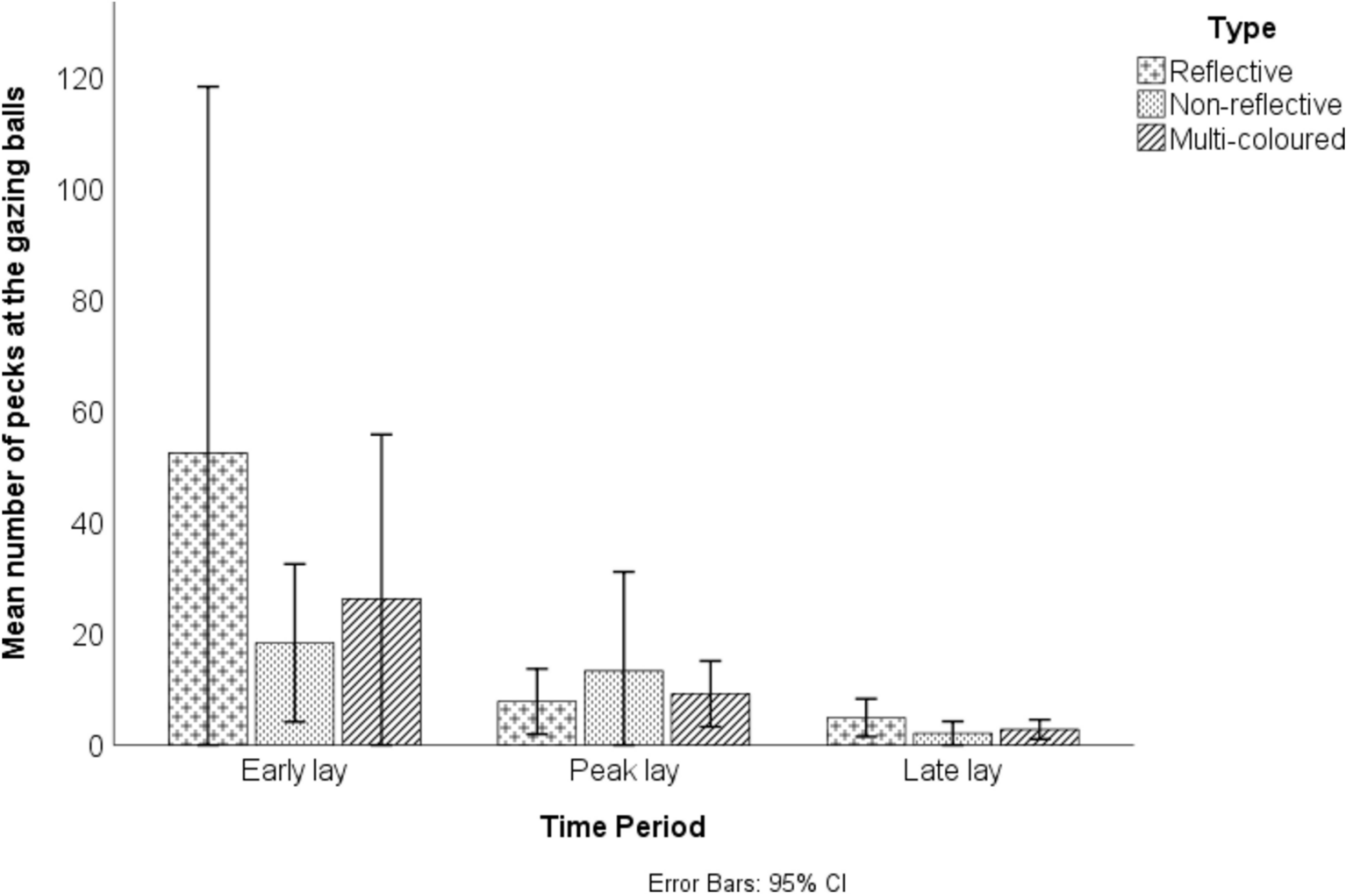
The mean number of pecks directed at the gazing ball pecking objects by broiler breeders over 5 min focal periods in early (weeks 25 and 27), peak (weeks 28–31) and late lay (weeks 44–46).

There was a significant effect of time period on the average number of pecks directed at the gazing balls during 5 min focal periods, with the highest interest in the gazing balls recorded during early lay (early lay EMM = 32.32, SE = 6.52; peak lay EMM = 10.12, SE = 4.97; late lay EMM = 3.35 SE = 5.51; F_4,89.01_, p = 0.01). Post-hoc comparison revealed a significantly higher number of pecks in early lay compared to peak and late lay (p < 0.05), with no significant difference between peak and late lay (p > 0.05). During this peak interest in early lay, there were an average of 52.38 (± 78.61) pecks directed at the reflective gazing ball, 18.33 (± 13.47) at the non-reflective gazing ball and 26.25 (± 35.24) at the multi-coloured gazing ball during the 5 min focal periods (Fig. 6). There was no significant difference between the types of pecking ball (either reflective, non-reflective or multi-coloured) but there was a large spread of data, suggesting that the number of pecks directed at the gazing balls during focal periods varied substantially depending on the observation (Fig. 6).

### Dustbathing material preference

**Fig. 7:**
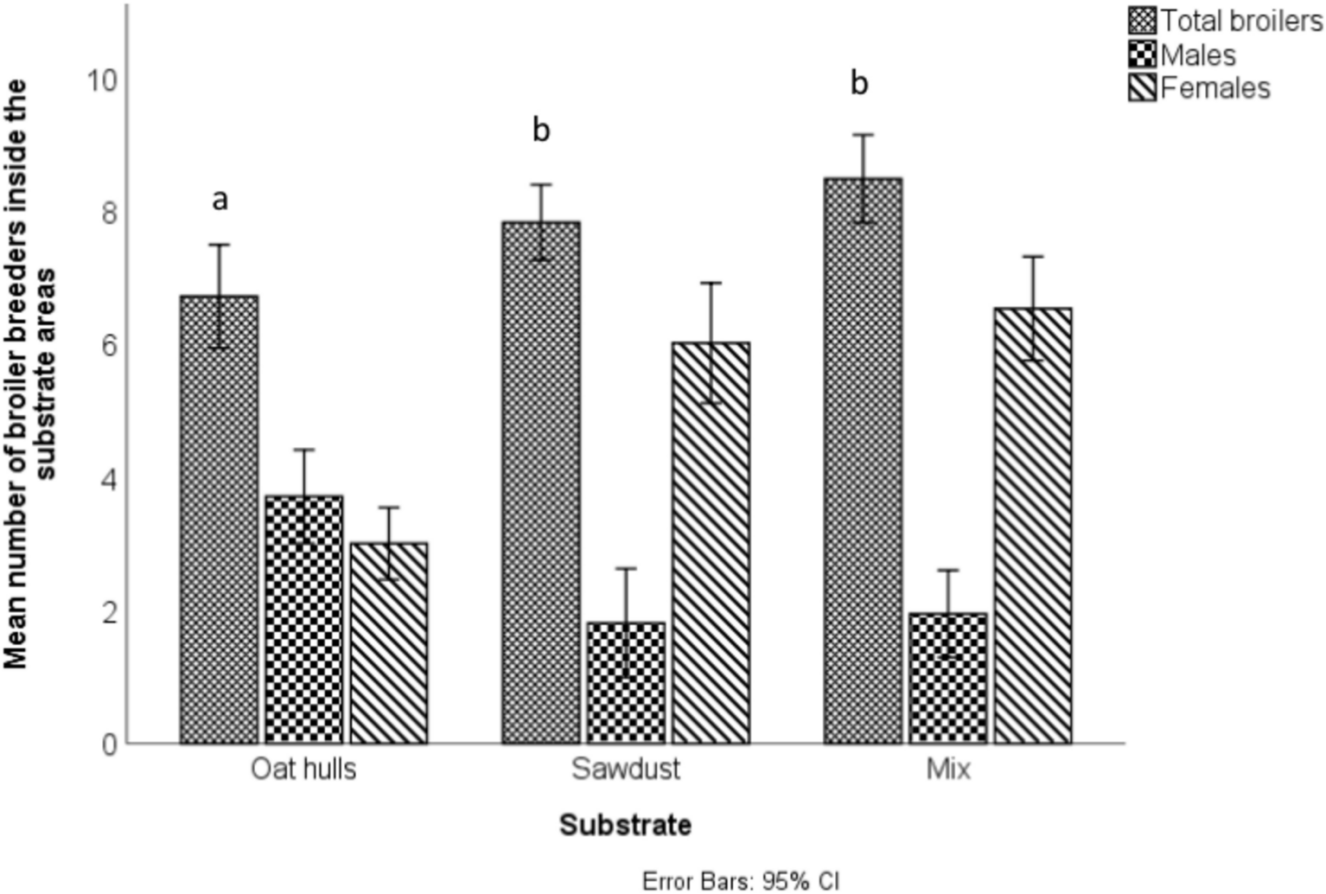
The average number of overall broiler breeders, males and females recorded in each substrate. Substrates were presented for one hour during weeks 28–31 and 43–46. Letters denote significance between substrates.

The litter was kept friable throughout the cycle and it was common to see dustbathing in the litter around the house. As the dustbathing substrates being evaluated were only provided for a short period, their novelty attracted a high number of birds which limited their capacity to be used for dustbathing in some instances. However, there were differences in the way the individual substrates were used and the way that males and females interacted with them. Substrate had a significant effect on the total number of birds recorded inside the substrate areas (N = 44, χ^2^(2) = 12.56, p = 0.002), with more breeders in the sawdust (p = 0.020) and mix (p < 0.001) compared to the oat hulls. However, this did not account for the number of birds standing outside the edge of the dustbathing areas and pecking into the oat hulls. The differences were also largely driven by the females as the highest number of males were recorded in the oat hulls (Fig. 7).

**Fig. 8:**
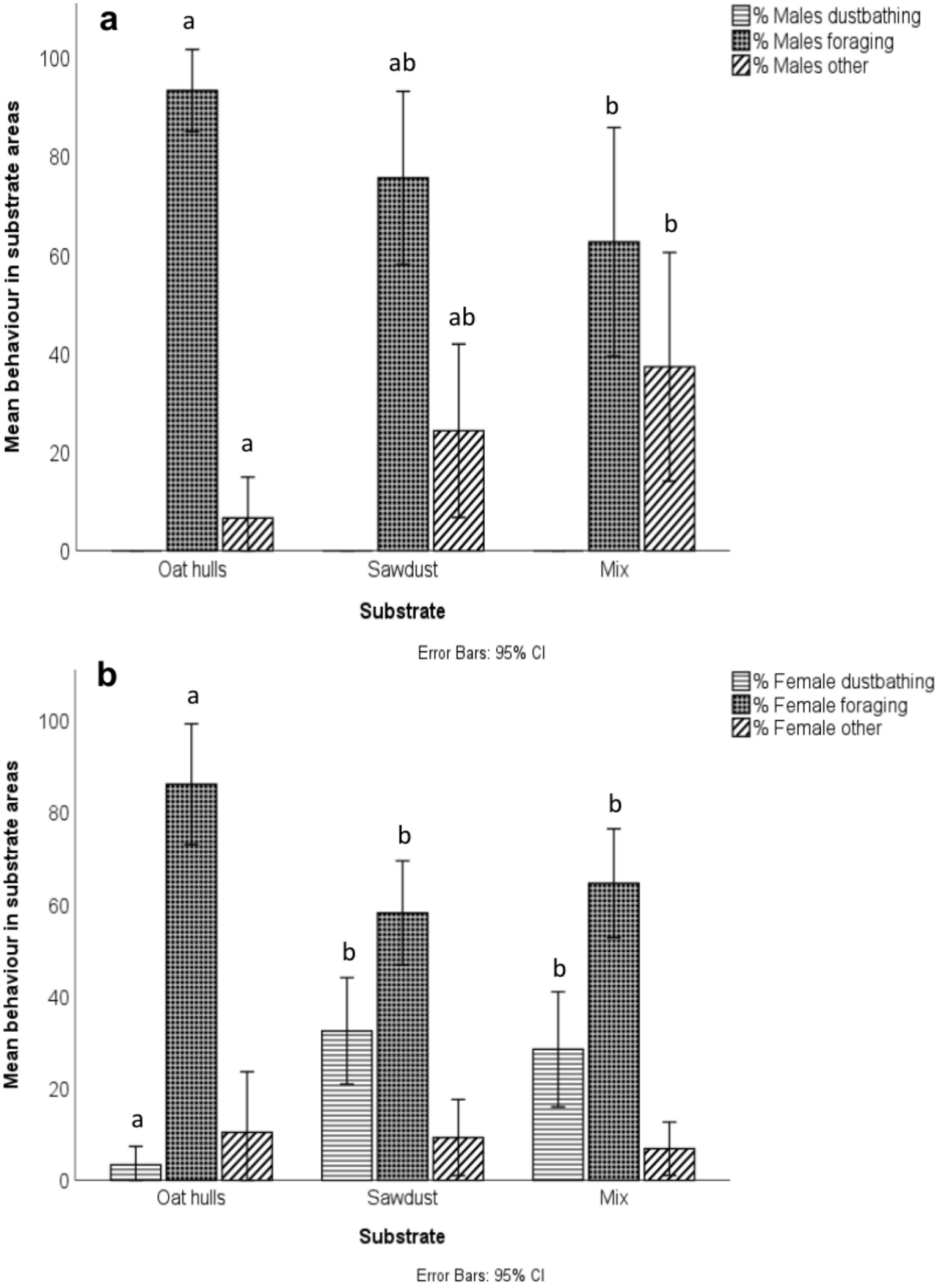
Male (**a**) and female (**b**) behaviour in dustbathing areas. Different letters denote significance for behaviours between susbtrates.

In oat hulls, males tended to move into the substrate areas first, with more males than females present on average until the 35 min scan (Supplementary Fig. 1). There were also a relatively high proportion of males initially in the sawdust and mix, considering the overall proportion of males in the house was approximately 8–10% (Supplementary Fig. 1). There was a significant effect of substrate on the proportion of broilers foraging among males (N = 42. χ^2^(2) = 6.23, p = 0.044; Fig. 8a) and females (N = 44, χ^2^(2) = 11.50, p = 0.003; Fig. 8b). A higher level of foraging was recorded in the oat hulls compared to the mix in males (p = 0.039) and in the oat hulls compared to the mix (p = 0.026) and sawdust (p = 0.001) in the females. Oat hulls also stimulated the lowest levels of dustbathing in females (N = 44, χ^2^(2) = 16.46, p < 0.001) compared to the mix (p = 0.002) and sawdust (p = 0.001; Fig. 8b). Interestingly, although males were seen dustbathing in the litter during the cycle, no males were observed dustbathing at all during the observations. Males were exclusively recorded as foraging or “other”, which was typically recorded when broilers were standing inactive (Fig. 8a).

### Overall house effects

#### Feather condition

**Fig. 9:**
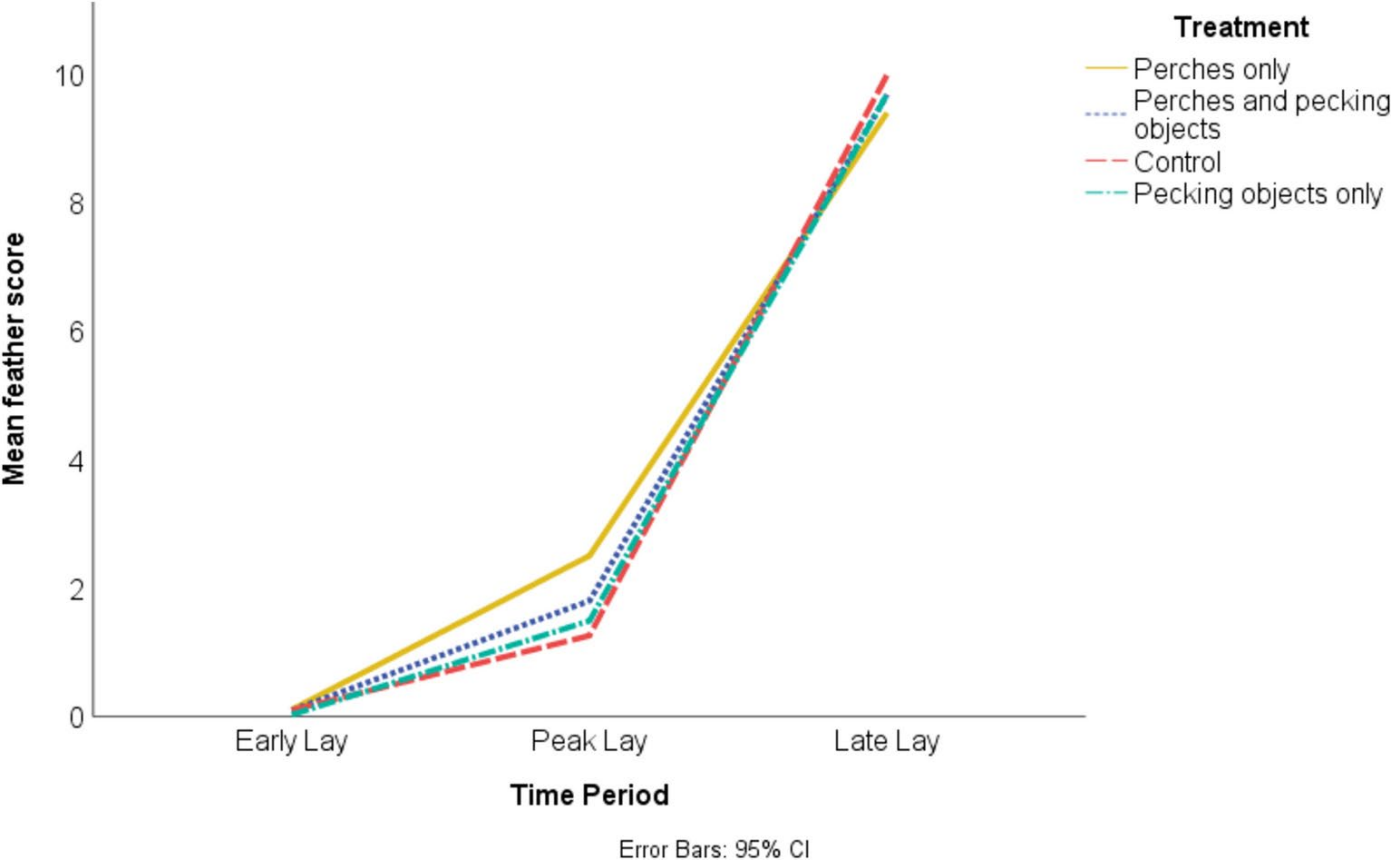
The mean feather score of broiler breeders in different houses containing either perches only (House 1), mixed enrichment of perches and pecking objects (House 2), no enrichment as a control (House 3) or pecking objects only (House 4). Feather score was recorded over the production cycle in each house during early (week 25), peak (week 31) and late lay (week 46).

Broiler breeders could receive a minimum score of 0 if their feather condition was perfect and a maximum score of 25 if all areas scored had > 50% feather loss with skin lesions. The maximum score recorded was 15; this was recorded four times in late lay only; once in the perches house, once in the mixed enrichment house and twice in the control house. Overall, the mean feather score was similar in each house (perches M = 4.00 ± 4.30; pecking objects M = 3.73 ± 4.49; mixed enrichment M = 3.86 ± 4.46; control M = 3.78 ± 4.64) but increased substantially over the production cycle (early lay M = 0.09 ± 0.31; peak lay M = 1.76 ± 1.60; late lay M = 9.68 ± 2.16). The largest numerical difference between the houses occurred in peak lay, with the lowest (best) mean feather score recorded in House 3 (unenriched control). This difference in mean feather score did not appear to continue into late lay (Fig. 9).

#### Production data

Overall data for weekly floor eggs, egg production and mortality are displayed in Table 1. There were numerical differences between the houses for % floor eggs, but all records were within expected commercial limits (floor eggs expected to stabilise around or below 2–3% after an initial peak of around 5%, depending on the breed; Moy Park Ltd, personal communication). The most crucial time for managing floor eggs is during early lay while pullets are learning to use nest boxes. The largest numerical difference in % floor eggs between the houses did occur during this time (Table 1; max weekly floor eggs occurred during early lay) but reduced towards the end of the production cycle. Overall, the highest egg production, highest % floor eggs and lowest mortality were seen in the house containing both forms of enrichment.

**Table 1:** Production data for broiler breeders over one production cycle.

	Overall mean ± SD	Min (weekly)	Max (weekly)
Weekly floor eggs (%)			
House 1 (Perches only)	0.66 ± 0.65	0	3.32
House 2 (Mixed enrichment)	0.82 ± 0.89	0.12	4.67
House 3 (Control)	0.52 ± 1.04	0.04	5.32
House 4 (Pecking object only)	0.44 ± 0.89	0.04	4.19
Weekly eggs produced (per hen)			
House 1 (Perches only)	4.63 ± 1.22	0	5.63
House 2 (Mixed enrichment)	4.77 ± 1.30	0.02	5.82
House 3 (Control)	4.64 ± 1.35	0.01	5.73
House 4 (Pecking object only)	4.63 ± 1.28	0.03	5.73
Average weekly mortality			
House 1 (Perches only)	16.4 ± 7.73	0	27
House 2 (Mixed enrichment)	9.12 ± 4.71	0	21
House 3 (Control)	15.1 ± 9.80	0	36
House 4 (Pecking object only)	13.1 ± 6.87	0	30

Data for four houses containing different enrichment conditions, either a combination of perch types, both perches and gazing balls (pecking enrichment), an unenriched control or gazing balls only. Descriptive data presented for each house, with mean and standard deviation (SD). Weekly floor eggs described any egg laid on the litter of the house, calculated as a percentage of the total weekly eggs produced. Weekly eggs produced per hen was calculated as the total eggs produced that week/total birds alive that week. Average egg data taken across weeks 23–63. Average weekly mortality is the average number of pullets dead per week (culled + found dead), between weeks 18–59.

## Discussion

The purpose of this study was to evaluate broiler breeder preference for environmental enrichments that had the potential to satisfy behavioural needs, reduce unwanted behaviours and increase the complexity of their housing. In commercial broiler breeder houses, we compared three types of perch (platform, round metal and round rubber-coated), pecking enrichment (reflective gazing ball, non-reflective gazing ball and multi-coloured reflective gazing ball) and dustbathing substrate (oat hulls, sawdust and a 50:50 mix). Breeders showed a sustained and clear preference for platform perches, with perches appearing to reach a comfortable maximum capacity once birds had 23 cm of linear space. The gazing balls appeared to attract pecking behaviour initially and no preference was detected between the types offered, although interest in them varied and waned over the production cycle. Widespread dustbathing in the friable house litter demonstrated the importance of dustbathing to breeders and there were some differences in the way the substrates were used, however the additional dustbathing areas proved unnecessary on this farm and more attractive as a novel foraging substrate. There were no clear negative impacts of increasing house complexity on egg production, mortality, feather score or the number of floor eggs.

Broiler breeders in this study showed a clear and consistent preference for platform perches made of plastic gridding over round steel and round rubber-coated perches. Although broiler chickens show a similar preference for platforms^16,17,^ breeders appear to be more physically agile than their offspring and may have preferred a rounded perch that would facilitate natural grasping behaviour, as has been reported in laying hens^8^. Similar research exploring perch preferences in commercially reared broiler breeders is sparse and it can be difficult to untangle the effects of height, material and other management factors. Vasdal et al.^15^ compared a selection of perch designs under commercial conditions in four flocks, finding a preference for steel square perches over round steel or round wooden perches when placed on the nesting platform. Plastic mushroom shaped perches added later then became the most preferred, although these were slightly higher which is often the most important factor affecting perch choice in domestic fowl^8,20,44^. Indeed, they found in a separate study that offered both 5 cm high plastic mushroom perches and 15 cm high steel plates on top of feeder lines, the feeder lines became the most preferred^14^. Brandes et al.^45^ demonstrated the wide range of perches available by comparing those already in place across 9 commercial flocks in Germany. Perch design and provision were diverse, with placement (raised platform or floor), material (plastic, wood or metal), shape (square, oval, round or mushroom shaped) and height (0–48 cm) varying widely. The most used perch was a 30 mm wooden rectangular perch fixed at 16 cm above the nesting platform in one barn, followed by wooden rectangular perches (48 cm high) and oval plastic perches (38–48 cm high) installed on the litter floor. A preference for square, rectangular or mushroom shaped perches likely point to the same inclination for a more stable horizontal surface that we found reflected in the preference for grid perches in the present study. The rubber-coated perches in this study were also used more than metal perches initially, which is consistent with breeders preferring a non-slip and more thermally stable roost. However, there was no difference in use between the two round perches by the end of the production cycle and it is possible that the rubber coating was not thick enough to offer a substantially different level of comfort^20^. The plastic gridding we used for grid perches was the same already in use for the nesting platforms, which could have led to a familiarity with that material. They had no previous experience of this gridding in the rearing house, which had no raised areas, and so a familiarity would only have formed when they moved to the laying house. Even if this did influence their perching behaviour outside of material preference alone, the plastic gridding is in use in UK breeder houses so a familiarity driven preference would still be advantageous if this material was used for perches in commercial housing.

Our perches differed significantly from other designs in that they were not fitted directly on to the nesting platform or floor but between roof support posts 53 cm higher than the nesting platform and 1 m from floor level. We found perches to have higher or comparable use to those previously reported in broiler breeders, particularly on the platform perches. Our average nighttime perching on platform perches was 3.2 birds/m or 74% occupancy, with breeders reaching 100% occupancy (based on 23 cm/bird) on platform perches in a quarter of nighttime hourly observations. Vasdal et al.^15^ reported an average of 0.90 birds/m on their most preferred perch (square steel) in the minutes before lights off. Similar to other studies (e.g.^45^) we found that perching levels were highest after lights off, however for direct comparison we recorded an average of 2.8 birds/m on platforms directly before lights off. When looking at breeders perching on metal plated feeder lines after dark, Vasdal et al.^14^ found a higher average of 5.3 birds/m. Brandes et al.^45^ report an overall perch occupancy of 2.07 birds/m and a higher occupancy on their most preferred perch of 4.86 birds/m in the dark period. The perches in this study were considerably higher than usually reported; 53 cm above the nesting platform and 1 m above floor level. For laying hens, a preference for roosts above 90 cm has been reported^46^ and EFSA recommend a height of at least 60 cm above the ground^20^. Although clearly an important and biologically relevant feature, increasing the height of perches does present additional risks of keel bone damage^12,47^ and could make them less accessible to older birds. Reports on perch use across the production cycle have been mixed, with perch use either remaining the same^14,15,45^ or reducing over time^12,13,48^. These results are difficult to compare due to the range in perch designs, heights and level of use, however we did see a decrease in perch use over time that can likely be attributed to declining agility. We did not assess keel bone injuries in the current study. There was also some concern that breeders would be unwilling to jump underneath a perch to access the nesting platform. However, in a comparison between the section with a perch and the neighbouring empty section, no difference was found in the number of broilers jumping up or down onto the platform. No issues were raised by the farmers and on-farm visits did not reveal any hesitation or distress at moving underneath the perches.

There is no legislation dictating space allowance for perching broiler breeders, although a perch length of 15 cm per hen is a requirement for laying hens (Council Directive 1999/74/EC). A linear allowance of 15 cm^14^ and 20 cm^13^ has been suggested based on breeder body size. Gebhardt-Heinrich et al.^48^ also compared different perch space allowances and found that more penned breeders perched with 14 cm/bird available compared to lower allowances, however there was no difference between 14 and 20 cm per bird. Brandes et al.^49^ suggested a larger space per bird of 21 cm for hens and 22 cm for cockerels based on body width measurements taken at 60 weeks, which is consistent with the recent EFSA recommendation^5^. We found that there was a drop off in perch use once 13 birds were perching, which would have given each bird 23 cm of linear space. This may reflect their level of comfort or the difficulty in accommodating more breeders jumping up once 4 birds/m was exceeded. Using shoulder width or the maximum observed number of birds perching (18 birds in our case, or 17 cm per bird) to determine maximum capacity may not accurately indicate comfortable capacity and could result in underestimating the necessary perching space.

We also tested breeder level of interest in three forms of suspended gazing ball to explore whether a pecking enrichment with reflective or colourful qualities would attract more sustained use. We found no significant difference between the number of pecks directed at gazing balls that were silver and reflective, silver and non-reflective, or multi-coloured and reflective. However, the gazing balls did attract considerable interest from broiler breeders. During 5-min focal periods, an average of 32.3 pecks at gazing balls were recorded during early lay, 10.1 during peak lay and 3.4 during late lay. It is worth noting that these observations were conducted over quite a narrow time window in the morning and may not be reflective of level of interaction with the gazing balls at other times. There was significant variation between videos, with apparent social contagion attracting numerous birds to peck in some cases, for example the maximum number of pecks recorded during a video was 403. The average levels recorded were comparable with our previous research with commercially housed broilers pecking at chain (average 70 pecks over 10-min focal period^28^) and significantly higher than interest shown towards other pecking enrichments in experimental conditions. For example, Arnould et al.^50^ report only 42 pecks directed to string by broilers across a 28 h period, while broilers given a visual enrichment of moving light spots recorded too few pecks across a 3-min focal period to be statistically analysed^51^. In laying hens, an average of 17 pecks at blocks of various materials were recorded over 30-min focal periods^52^, or 0.15 pecks per hour at concrete pecking blocks^53^. These studies were all performed in pens, so it is likely that the chickens will become accustomed to the enrichments faster than in commercial conditions where they encounter them less frequently. Regardless, we did see less direct interaction as birds aged, which is a common issue with environmental additions^54^. Our metric for measuring interest in the gazing balls may have also underestimated interaction with the enrichment. The main feature of the gazing balls was their visual interest as a reflective or colourful surface that gave the chickens a moving surface to look at. During observations it was difficult to determine when a passing breeder was looking into the gazing ball, unless it was for a prolonged period, which meant counting the number of pecks was a more direct measure of interaction. However, vision is considered the dominant sense in poultry, they have a wide field of view and better colour vision than humans^55–57^. It is therefore likely that their visual perception and interest in the gazing balls was the most important feature of the enrichment. In addition to the level of direct use recorded, increasing the complexity of an environment could itself promote exploration and help animals acquire information about their surroundings^58^. For example, calves show more exploratory behaviour in pens with multiple objects (brushes, rope, strings, hay net, teats) compared to pens containing one object at a time^59^.

The house litter on this farm was deep and generally kept in good condition throughout the cycle which facilitated a much higher level of general dustbathing on the floor than we expected. Dustbathing is clearly an important behaviour to broiler breeders and large groups of birds dustbathing together was common. This meant that although the novelty of the experimental dustbathing areas attracted a high number of birds, they were generally superfluous as a method of facilitating dustbathing. However, as anticipated we did find that higher levels of dustbathing were observed in the non-nutritive sawdust compared to oat hulls and 50:50 mix. The least number of breeders were also recorded inside the oat hulls areas, although we observed that a large number of birds would stand outside the areas and peck into the substrate, which would seem to confirm the suggestion that oat hulls were primarily used as a source of forage. We only provided dustbathing areas for brief periods of 1 h, but they were almost always completely cleared of substrate by the end of that time, which means that they would require an impractical level of maintenance. This is in considerable contrast to the use of dustbathing areas in broiler housing, where substrates were typically topped up once a week and stimulated high levels of dustbathing^39,40^. There was an interesting difference in the way males and females used the dustbathing areas, with a large number of males entering the dustbathing areas initially, before more females entered and the males moved away. However, we observed no males dustbathing in any substrates (although males were observed dustbathing in the house litter). This early access to the substrates may reflect a higher motivation in males to find food sources, for example male broilers walked faster along a runway to reach a food reward than females, despite being heavier^60^. Although normal courtship behaviours are reduced in broiler breeders^61,62^, food also plays a role in mating behaviour which could have motivated males to access a novel substrate. Males calling females to a real or false food source (e.g. an insect or a twig) to initiate mating behaviour is a common initial element of poultry courtship called “tidbitting”^61,63,64^. Although outside the scope of this study, male response to a novel food source would be an interesting area to explore further.

Although not the primary goal of this study, it was important that environmental enrichments were practical and had no negative impact on productivity for them to be suitable for commercial housing. The enrichments in this study were only installed in three houses with one unenriched control house and therefore the impact of environmental complexity is confounded with the potential effect of different houses. However, the houses were identical and generally maintained to produce homogenous flocks, so we cautiously interpret the results with reference to the enrichments provided. We found no negative impact of perches, dustbathing areas or suspended gazing balls being present in the houses. All production results were within normal commercial limits for this producer. There was some concern that perches would lead to an increase in floor eggs, as reported in aviary systems by Gebhardt-Henrich et al.^12^. However, in our study the peak in floor eggs seen during early lay was highest in the control house. Better feather scores were also seen in the control house, although this effect was only seen early in the cycle. Overall, the highest egg production, highest % floor eggs and lowest mortality was seen in the house containing all enrichments.

Our method of installing perches meant they could be higher than if they had been placed directly on the nesting platform, and they did not take up available space on the nesting platform or block nest access. The 12 perches installed per house equated to 4.5 m of perch space per 1000 birds, which is in excess of the recommendations for broiler chickens of 2 m per 1000 birds [65]. A maximum of 52 perches could have been installed if all the space between posts was used in each house, providing 156 m of perching space or 20 m per 1000 birds. Although it would be preferred for all breeders to be able to perch simultaneously, providing access to platform perches would satisfy the EFSA^5^ recommendation that breeders be provided with both elevated platform areas (i.e. the nesting platform) and perches. Within the restrictions of existing house design this provision of higher perches off the nesting platform would satisfy the motivation to roost at height for those physically able and reduce the stocking density of nesting platforms at night^65^.

## Conclusions

In a comparison between platform perches made of plastic gridding, round metal perches and round rubber-coated perches installed 53 cm above the nesting platforms and 1 m from floor level, commercially housed broiler breeders showed a clear and sustained preference for grid perches. Perching levels were generally high on the platform perches, especially at night, and we found a drop off in the number of breeders occupying the perch once 23 cm of linear space per bird had been reached. Suspended gazing balls used to increase environmental complexity and attract pecking behaviour drew significant interest from the breeders, although this did reduce as birds aged. Whether the gazing balls were reflective, non-reflective or colourful did not impact their level of use. We also tested oat hulls, sawdust and a 50:50 mix as materials provided to stimulate dustbathing. However, good quality litter facilitated high levels of dustbathing on the house floor, which meant that the additional novel substrates were largely used for exploration and foraging. There were no obvious impacts of any additions on health and productivity measures and the farmer and producer were satisfied with the practicality of platform perches and gazing balls as environmental enrichments that could be installed in commercial housing.

## Supplementary Information

Supplementary Figure 1.

## Data Availability

The data that support the findings of this study are not openly available due to reasons of commercial sensitivity and are available from the corresponding author upon reasonable request.

## References

[1] Riber, A. B., Van De Weerd, H., De Jong, I. & Steenfeldt, S. Review of environmental enrichment for broiler chickens. *Poult. Sci.* **97**, 378–396 (2018). 29211895 10.3382/ps/pex344

[2] Campbell, D., De Haas, E. & Lee, C. A review of environmental enrichment for laying hens during rearing in relation to their behavioral and physiological development. *Poult. Sci.* **98**, 9–28 (2019). 30107615 10.3382/ps/pey319 PMC6347129

[3] Jacobs, L. et al. Enhancing their quality of life: environmental enrichment for poultry. *Poult. Sci.* **102**, 102233 (2023). 36351344 10.1016/j.psj.2022.102233 PMC9647224

[4] *Better Chicken Commitment: The Policy*. betterchickencommitment.com (2024).

[5] EFSA Ahaw Panel (EFSA Panel on Animal Health and Animal Welfare). Scientific opinion on the welfare of broilers on farm. *EFSA J.*10.2903/j.efsa.2023.7788 (2023).

[6] Riber, A. B., de Jong, I. C., van de Weerd, H. A. & Steenfeldt, S. Environmental enrichment for broiler breeders: An undeveloped field. *Front. Vet. Sci.* **4**, 86 (2017). 28649569 10.3389/fvets.2017.00086 PMC5465254

[7] Newberry, R. C., Estevez, I. & Keeling, L. J. Group size and perching behaviour in young domestic fowl. *Appl. Anim. Behav. Sci.* **73**, 117–129 (2001). 11358609 10.1016/s0168-1591(01)00135-6

[8] Schrader, L. & Müller, B. Night-time roosting in the domestic fowl: The height matters. *Appl. Anim. Behav. Sci.* **121**, 179–183 (2009).

[9] Council of Europe. *Recommendation concerning domestic fowl (Gallus Gallus).* https://www.coe.int/t/e/legal_affairs/legal_co-operation/biological_safety_and_use_of_animals/farming/Rec%20fowl%20E.asp (1995).

[10] Lower Saxony Federal Ministry for Food and Agriculture. *Minimum requirements for the keeping of broiler breeders*. https://www.umwelt-online.de/recht/natursch/laender/nds/masthuehnerelt.htm#:~:text=1.1%20Die%20Besatzdichte%20darf%20maximal,ihrer%20Ruhestellung%20ohne%20Verletzungsgefahr%20erm%C3%B6glicht (2015).

[11] Swiss Federal Act on Animal Welfare. *Animal Welfare Ordinance*. https://www.globalanimallaw.org/downloads/database/national/switzerland/TSchV-2008-EN-455.1-2011.pdf. (2008).

[12] Gebhardt-Henrich, S. G., Toscano, M. J. & Würbel, H. Use of aerial perches and perches on aviary tiers by broiler breeders. *Appl. Anim. Behav. Sci.* **203**, 24–33 (2018).

[13] Mens, A. J. & van Emous, R. A. Broiler breeders roosted more on slats than on perches during the laying period. *Appl. Anim. Behav. Sci.* **246**, 105531 (2022).

[14] Vasdal, G., Gebhardt-Henrich, S., Tahamtani, F. & Kittelsen, K. Effect of perch access on perching, health and production outcomes in commercial broiler breeder flocks. *Poult. Sci.* **101**, 102160 (2022). 36167022 10.1016/j.psj.2022.102160 PMC9516462

[15] Vasdal, G., Gebhardt-Henrich, S., Tahamtani, F. & Kittelsen, K. Perch use in commercial broiler breeders–Preference for perch material and effect of age. *Appl. Anim. Behav. Sci.* **253**, 105680 (2022).

[16] Norring, M., Kaukonen, E. & Valros, A. The use of perches and platforms by broiler chickens. *Appl. Anim. Behav. Sci.* **184**, 91–96 (2016).

[17] Bailie, C. L., Baxter, M. & O’Connell, N. E. Exploring perch provision options for commercial broiler chickens. *Appl. Anim. Behav. Sci.* **200**, 114–122 (2018).

[18] Struelens, E. et al. Perch width preferences of laying hens. *Br. Poult. Sci.* **50**, 418–423 (2009). 19735010 10.1080/00071660903110885

[19] Pickel, T., Scholz, B. & Schrader, L. Perch material and diameter affects particular perching behaviours in laying hens. *Appl. Anim. Behav. Sci.* **127**, 37–42 (2010).

[20] EFSA Ahaw Panel (EFSA Panel on Animal Health and Animal Welfare). Scientific opinion on welfare aspects of the use of perches for laying hens. *EFSA J.* **13**, 70. 10.2903/j.efsa.2015.4131 (2015).

[21] Dawkins, M. S. Time budgets in red junglefowl as a baseline for the assessment of welfare in domestic fowl. *Appl. Anim. Behav. Sci.* **24**, 77–80 (1989).

[22] Newberry, R. C. Exploratory behaviour of young domestic fowl. *Appl. Anim. Behav. Sci.* **63**, 311–321 (1999). 10.1016/s0168-1591(01)00135-6 11358609

[23] Olsson, P., Lind, O. & Kelber, A. Bird colour vision: behavioural thresholds reveal receptor noise. *J. Exp. Biol.* **218**, 184–193 (2015). 25609782 10.1242/jeb.111187

[24] Chamove, A. S. Environmental enrichment: a review. *Anim. Technol.* **40**, 155–178 (1989).

[25] Jones, B. R. Role of comparative psychology in the development of effective environmental enrichment strategies to improve poultry welfare. *Int. J. Comp. Psychol.*10.46867/C4M01G (2002).

[26] Leone, E. & Estévez, I. Economic and welfare benefits of environmental enrichment for broiler breeders. *Poult. Sci.* **87**, 14–21 (2008). 18079444 10.3382/ps.2007-00154

[27] Lourenço da Silva, M. I. et al. Behaviour and animal welfare indicators of broiler chickens housed in an enriched environment. *PloS ONE* **16**, e0256963 (2021). 34570782 10.1371/journal.pone.0256963 PMC8476007

[28] Baxter, M. & O’Connell, N. E. Does grouping environmental enrichments together affect the way they are used by commercially housed broiler chickens?. *Appl. Anim. Behav. Sci.* **210**, 52–59 (2019).

[29] Bell, D., Adams, C. & Gvaryahu, G. Environment enrichment devices for caged laying hens. *J. Appl. Poult. Res.* **7**, 19–26 (1998).

[30] Bailie, C. & O’Connell, N. The influence of providing perches and string on activity levels, fearfulness and leg health in commercial broiler chickens. *Animal* **9**, 660–668 (2015). 25440236 10.1017/S1751731114002821

[31] Guinebretière, M. et al. Effects of management strategies on non-beak-trimmed laying hens in furnished cages that were reared in a non-cage system. *Animals* **10**, 399 (2020). 32121241 10.3390/ani10030399 PMC7142790

[32] Clarke, C. H. & Jones, R. B. Responses of adult laying hens to abstract video images presented repeatedly outside the home cage. *Appl. Anim. Behav. Sci.* **67**, 97–110 (2000). 10719193 10.1016/s0168-1591(99)00107-0

[33] Rheingold, H. L. & Hess, E. H. The chick’s “preference” for some visual properties of water. *J. Comp. Physiol. Psychol.* **50**, 417 (1957). 13481175 10.1037/h0046108

[34] Hunt, G. L. Jr. & Smith, W. J. Pecking and initial drinking responses in young domestic fowl. *J. Comp. Physiol. Psychol.* **64**, 230 (1967). 6050567 10.1037/h0088041

[35] Horn, G. Pathways of the past: the imprint of memory. *Nat. Rev. Neurosci.* **5**, 108–120 (2004). 14735114 10.1038/nrn1324

[36] Vestergaard, K. Dust-bathing in the domestic fowl—diurnal rhythm and dust deprivation. *Appl. Anim. Ethol.* **8**, 487–495 (1982).

[37] Van Liere, D. The significance of fowls’ bathing in dust. *Anim. Welf.* **1**, 187–202 (1992).

[38] Widowski, T. M. & Duncan, I. J. Working for a dustbath: are hens increasing pleasure rather than reducing suffering?. *Appl. Anim. Behav. Sci.* **68**, 39–53 (2000). 10771314 10.1016/s0168-1591(00)00088-5

[39] Baxter, M., Bailie, C. & O’Connell, N. An evaluation of potential dustbathing substrates for commercial broiler chickens. *Animal* **12**, 1933–1941 (2018). 29271337 10.1017/S1751731117003408

[40] Baxter, M., Bailie, C. L. & O’Connell, N. E. Evaluation of a dustbathing substrate and straw bales as environmental enrichments in commercial broiler housing. *Appl. Anim. Behav. Sci.* **200**, 78–85 (2018).

[41] Scholey, D., Marshall, A. & Cowan, A. Evaluation of oats with varying hull inclusion in broiler diets up to 35 days. *Poult. Sci.* **99**, 2566–2572 (2020). 32359592 10.1016/j.psj.2019.12.043 PMC7597440

[42] Morrissey, K. et al. The effect of dietary alterations during rearing on feather condition in broiler breeder females. *Poult. Sci.* **93**, 1636–1643 (2014). 24864283 10.3382/ps.2013-03822

[43] Arrazola, A. et al. The effect of alternative feeding strategies for broiler breeder pullets: 1. Welfare and performance during rearing. *Poult. Sci.* **98**, 3377–3390 (2019). 31001626 10.3382/ps/pez170 PMC6698192

[44] Brendler, C., Kipper, S. & Schrader, L. Vigilance and roosting behaviour of laying hens on different perch heights. *Appl. Anim. Behav. Sci.* **157**, 93–99 (2014).

[45] Brandes, A., Giersberg, M., Kemper, N. & Spindler, B. Provision of perches and their use by broiler breeders on the basis of a case study. *Eur. Poult. Sci. Arch. Geflügelkund.*10.1399/eps.2020.311 (2020).

[46] Brendler, C. & Schrader, L. Perch use by laying hens in aviary systems. *Appl. Anim. Behav. Sci.* **182**, 9–14 (2016).

[47] Sandilands, V., Moinard, C. & Sparks, N. Providing laying hens with perches: fulfilling behavioural needs but causing injury?. *Br. Poult. Sci.* **50**, 395–406 (2009). 19735008 10.1080/00071660903110844

[48] Gebhardt-Henrich, S., Toscano, M. J. & Würbel, H. Perch use by broiler breeders and its implication on health and production. *Poult. Sci.* **96**, 3539–3549 (2017). 28938782 10.3382/ps/pex189

[49] Brandes, A. G., Spindler, B., Giersberg, M. F. & Kemper, N. Feed space allowance and perch design criteria for broiler breeders determined by biometric data. *Vet. Sci.* **9**, 350 (2022). 35878367 10.3390/vetsci9070350 PMC9321206

[50] Arnould, C., Bizeray, D., Faure, J. & Leterrier, C. Effects of the addition of sand and string to pens on use of space, activity, tarsal angulations and bone composition in broiler chickens. *Anim. Welf.* **13**, 87–94 (2004).

[51] Bizeray, D., Estevez, I., Leterrier, C. & Faure, J. Effects of increasing environmental complexity on the physical activity of broiler chickens. *Appl. Anim. Behav. Sci.* **79**, 27–41 (2002).

[52] Dixon, L., Duncan, I. & Mason, G. The effects of four types of enrichment on feather-pecking behaviour in laying hens housed in barren environments. *Anim. Welf.* **19**, 429–435 (2010).

[53] Holcman, A., Gorjanc, G. & Štuhec, I. Porous concrete block as an environmental enrichment device increases activity of laying hens in cages. *Poult. Sci.* **87**, 1714–1719 (2008). 18753437 10.3382/ps.2008-00113

[54] Tarou, L. R. & Bashaw, M. J. Maximizing the effectiveness of environmental enrichment: Suggestions from the experimental analysis of behavior. *Appl. Anim. Behav. Sci.* **102**, 189–204 (2007).

[55] Prescott, N., Wathes, C. M. & Jarvis, J. Light, vision and the welfare of poultry. *Anim. Welf.* **12**, 269–288 (2003).

[56] Lewis, P. D. & Morris, T. R. Poultry and coloured light. *World Poult. Sci. J.* **56**, 189–207 (2006).

[57] Akyüz, H. Ç. & Onbaşilar, E. Light wavelength on different poultry species. *World Poult. Sci. J.* **74**, 79–88 (2018).

[58] Veissier, I., Lesimple, C., Brunet, V., Aubé, L. & Botreau, R. Rethinking environmental enrichment as providing opportunities to acquire information. *Animal* **18**, 101251 (2024). 39137615 10.1016/j.animal.2024.101251

[59] Zhang, C., Juniper, D. T. & Meagher, R. K. Effects of physical enrichment and pair housing before weaning on growth, behaviour and cognitive ability of calves after weaning and regrouping. *Appl. Anim. Behav. Sci.* **249**, 105606 (2022).

[60] Bokkers, E. A. & Koene, P. Sex and type of feed effects on motivation and ability to walk for a food reward in fast growing broilers. *Appl. Anim. Behav. Sci.* **79**, 247–261 (2002). 10.1016/j.beproc.2004.03.015 15240050

[61] Millman, S., Duncan, I. & Widowski, T. Male broiler breeder fowl display high levels of aggression toward females. *Poult. Sci.* **79**, 1233–1241 (2000). 11020065 10.1093/ps/79.9.1233

[62] De Jong, I., Wolthuis-Fillerup, M. & Van Emous, R. Development of sexual behaviour in commercially-housed broiler breeders after mixing. *Br. Poult. Sci.* **50**, 151–160 (2009). 19373714 10.1080/00071660802710124

[63] Gyger, M. & Marler, P. Food calling in the domestic fowl, Gallus gallus: the role of external referents and deception. *Anim. Behav.* **36**, 358–365 (1988).

[64] Zuk, M. et al. The role of male ornaments and courtship behavior in female mate choice of red jungle fowl. *Am. Nat.* **136**, 459–473 (1990).

[65] Red Tractor Certified Standards. Chicken Standards: Broilers and Poussin. https://redtractorassurance.org.uk/wp-content/uploads/2021/08/Chicken-Broiler-and-Pousin-Standards.pdf (2019).

