# Supplementary material for: Developing appropriate environmental enrichment strategies for broiler breeders
Source: Sci Rep. 2025 Mar 21;15:9738. doi: 10.1038/s41598-025-89691-w (PMC11928546; doi:10.1038/s41598-025-89691-w)
Supplement: Supplementary file 1 — Supplementary Figure 1. [file 41598_2025_89691_MOESM1_ESM.pdf]

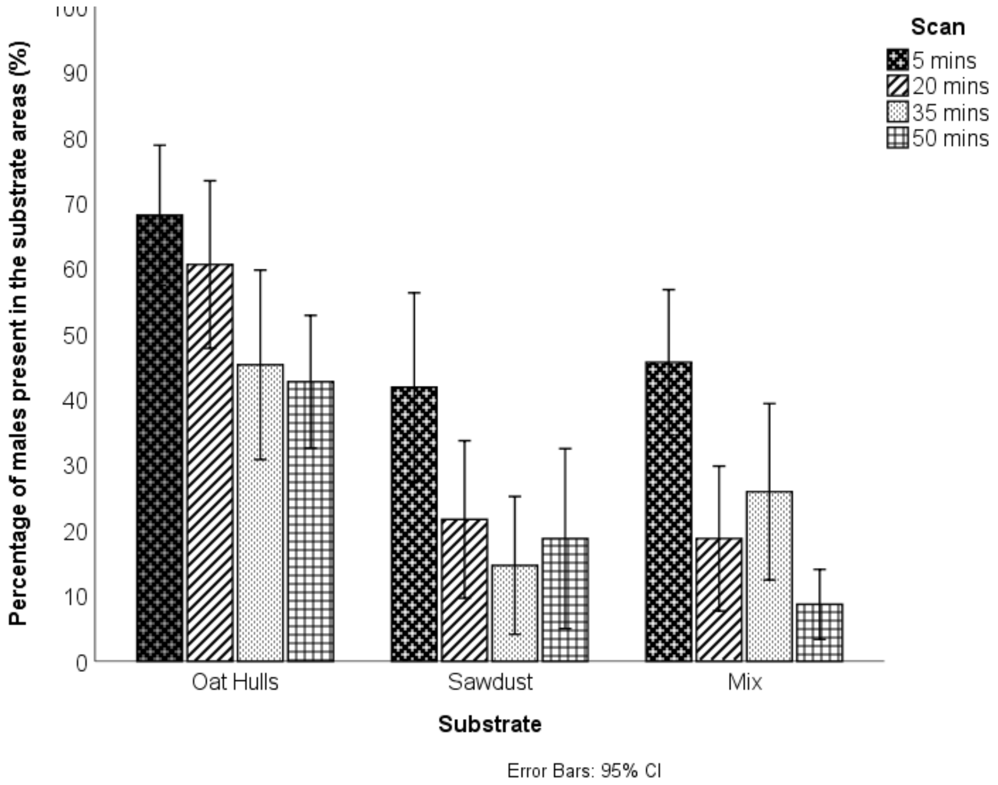

Supplementary Figure 1. The proportion of broiler breeders in the dustbathing area that are male over the focal period.
